# Histological and toxicological evaluation, in rat, of a P-glycoprotein inducer and activator: 1-(propan-2-ylamino)-4-propoxy-9*H*-thioxanthen-9-one (TX5)

**DOI:** 10.17179/excli2019-1675

**Published:** 2019-08-27

**Authors:** Carolina Rocha-Pereira, Vera Silva, Vera Marisa Costa, Renata Silva, Juliana Garcia, Salomé Gonçalves-Monteiro, Margarida Duarte-Araújo, Alice Santos-Silva, Susana Coimbra, Ricardo Jorge Dinis-Oliveira, Catarina Lopes, Paula Silva, Solida Long, Emília Sousa, Maria de Lourdes Bastos, Fernando Remião

**Affiliations:** 1 UCIBIO/REQUIMTE, Laboratório de Toxicologia, Departamento de Ciências Biológicas, Faculdade de Farmácia, Universidade do Porto, Rua Jorge Viterbo Ferreira, 228, 4050-313 Porto, Portugal; 2 CITAB - Centre for the Research and Technology of Agro-Environmental and Biological Sciences, Department of Agronomy, University of Trás-os-Montes e Alto Douro, 5001-801 Vila Real, Portugal; 3 LAQV/REQUIMTE, Laboratório de Farmacologia, Departamento de Ciências do Medicamento, Faculdade de Farmácia, Universidade do Porto, Rua Jorge Viterbo Ferreira, 228, 4050-313 Porto, Portugal; 4 LAQV/REQUIMTE, Departamento de Imuno-Fisiologia e Farmacologia, Instituto de Ciências Biomédicas de Abel Salazar (ICBAS), Universidade do Porto, Rua Jorge Viterbo Ferreira, 228, 4050-313 Porto, Portugal; 5 UCIBIO/REQUIMTE, Laboratório de Bioquímica, Departamento de Ciências Biológicas, Faculdade de Farmácia, Universidade do Porto, Rua Jorge Viterbo Ferreira, 228, 4050-313 Porto, Portugal; 6 Instituto de Investigação e Formação Avançada em Ciências e Tecnologias Saúde (IINFACTS), Departamento de Ciências, Instituto Universitário de Ciências da Saúde (IUCS-CESPU), Rua Central de Gandra, 1317, 4585-116 Gandra, Portugal; 7 Departamento de Saúde Pública e Ciências Forenses e Educação Médica, Faculdade de Medicina, Universidade do Porto, Alameda Prof. Hernâni Monteiro, 4200-319 Porto, Portugal; 8 Molecular Oncology and Viral Pathology Group, Centro de Investigação do IPO-Porto; 9 Departamento de Microscopia, Laboratório de Histologia e Embriologia, Instituto de Ciências Biomédicas de Abel Salazar (ICBAS), Universidade do Porto, Rua Jorge Viterbo Ferreira, 228, 4050-313 Porto, Portugal; 10 CIIMAR, Laboratório de Química Orgânica e Farmacêutica, Departamento de Ciências Químicas, Faculdade de Farmácia, Universidade do Porto, Rua Jorge Viterbo Ferreira, 228, 4050-313 Porto, Portugal

**Keywords:** thioxanthones, oxidative stress, P-glycoprotein, toxicological biomarkers, peripheral toxicity

## Abstract

P-glycoprotein (P-gp) is an ATP-binding cassette transporter involved in the efflux of numerous compounds that influences the pharmacokinetics of xenobiotics. It reduces intestinal absorption and exposure of target cells to toxicity. Thioxanthones are compounds able to induce and/or activate P-gp *in vitro*. Particularly, 1-(propan-2-ylamino)-4-propoxy-9*H*-thioxanthen-9-one (TX5) behaves as a P-gp inducer and activator *in vitro*. The aims of this study were: i) to perform a histological characterization, by testing a single high dose of TX5 [30 mg/kg, body weight (b.w.), gavage], administered to Wistar Han rats, 24 hours after administration; and ii) to perform both a complete histological characterization and a preliminary safety evaluation, in distinct target organs, 24 hours after administration of a single lower dose of TX5 (10 mg/kg, b.w., gavage) to Wistar Han rats. The results showed a relevant histological toxicity for the higher dose of TX5 administered (30 mg/kg, b.w.), manifested by extensive hepatic necrosis and splenic toxicity (parenchyma with hyperemia, increased volume of both white and red pulp, increased follicles marginal zone). Moreover, in the kidneys, a slight hyperemia and tubular edema were observed in TX5-treated animals, as well as an inflammation of the small intestine. On the contrary, for the lower tested dose (10 mg/kg, b.w.), we did not observe any relevant histological toxicity in the evaluated organs. Additionally, no significant differences were found in the ATP levels between TX5-exposed and control animals in any of the evaluated organs, with the exception of the intestine, where ATP levels were significantly higher in TX5-treated rats. Similarly, TX5 caused a significant increase in the ratio GSH/GSSG only in the lungs. TX5 (10 mg/kg, b.w.) did not induce any change in any of the hematological and biochemical circulating evaluated parameters. However, TX5 was able to significantly reduce the activated partial thromboplastin time, without affecting the prothrombin time. The urine biochemical analysis revealed a TX5-mediated increase in both creatinine and sodium. Taken together, our results show that TX5, at a dose of 10 mg/kg, does not induce considerable toxicity in the biological matrices studied. Given this adequate safety profile, TX5 becomes a particularly interesting compound for *ex vivo* and *in vivo* studies, regarding the potential for induction and activation of P-gp at the intestinal barrier.

## Graphical Abstract


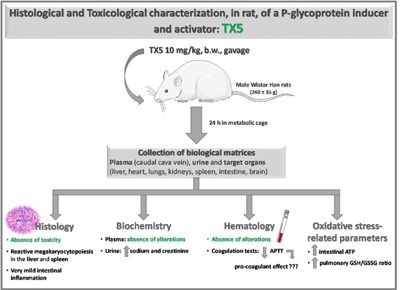


## Abbreviations

ABC: ATP-binding cassette

APTT: activated partial thromboplastin time

ATP: adenosine triphosphate

BSA: bovine serum albumin

b.w.: body weight

CSF: cerebrospinal fluid

DTNB: 5,5′-dithiobis(2-nitrobenzoic acid)

EDTA: ethylenediaminetetraacetic acid

GFR: glomerular filtration rate

GR: glutathione reductase

GSH: reduced glutathione

GSSG: oxidized glutathione

Hb: hemoglobin

H&E: hematoxylin-eosin

HPLC-DAD: high-performance liquid chromato-graphy with diode-array detection

i.p.: intraperitoneally

MCH: mean cell hemoglobin

MCHC: mean cell hemoglobin concentration

MCV: mean cell volume

MDR: multidrug resistance 

MPT: mitochondrial permeability transition

MPV: mean platelet volume

NADPH: nicotinamide phosphate adenine dinucleotide

PALS: periarterial lymphatic sheath

PAS: periodic acid-schiff

PCT: plateletcrit

PDW: platelet distribution width

P-gp: P-glycoprotein

PLT: platelet

PT: prothrombin time

RDW: red cell distribution width

ROW: relative organ weight

SGLT2: sodium/glucose co-transporter 2

tGSH : total glutathione

TNB: 5'-thio-2-nitrobenzoic acid

TT: thrombin time

TXs: thioxanthones

TX5: 1-(propan-2-ylamino)-4-propoxy-9*H*-tioxanthen-9-one

WBC: white blood cell

## Introduction

Thioxanthones (TXs) are heterocyclic compounds with several reported biological activities and therapeutic potential, namely those related to antitumor activity and P-glycoprotein (P-gp) modulation potential (Paiva et al., 2013[27]). ATP-binding cassette (ABC) carriers are efflux pumps mainly located at the plasma membrane of barrier tissues, such as the intestine, where they affect the pharmaco/toxicokinetics of both endobiotics and xenobiotics. For this reason, ABC proteins are involved in the multidrug resistance (MDR) phenomenon, being responsible for the extrusion of many unrelated therapeutically important drugs, including anticancer agents. On the other hand, ABC proteins, due to their efflux function, are important to maintain cellular homeostasis and to detoxify potentially toxic substances by avoiding their accumulation inside the cells (DeGorter et al., 2012[9]; Silva et al., 2015[41]). P-gp belongs to the ABCB subfamily of carriers and is one of the most studied ABC proteins, being involved in drug efficacy and toxicity. Given the involvement of ABC carriers, particularly P-gp, in the defense mechanism against toxic substances, as well as in the MDR phenotype, they play a relevant role in physiological, pharmacological and toxicological fields (Couture et al., 2006[8]; Silva et al., 2015[41]). As such, there is much interest in modulating ABC efflux function. Compounds interacting with ABC transporters can act as substrates, inhibitors, inducers or activators, but one compound can also have overlapping modes of action (Gameiro et al., 2017[15]; Wessler et al., 2013[49]). Several *in vitro* and *in vivo* studies have shown that both the expression levels and the activity of ABC transporters can be modulated by inducers (Dinis-Oliveira et al., 2006[10]; Silva et al., 2011[37]; Silva et al., 2013[38]) and activators (Martins et al., 2019[26]; Silva et al., 2015[39]; Silva et al., 2014[40]; Vilas-Boas et al., 2013[47]). The inducing and activating effects have been shown to be triggered by (thio)xanthonic compounds, protecting different cell models against paraquat-mediated toxicity (Martins et al., 2019[26]; Silva et al., 2015[39]; Silva et al., 2014[40]). Particularly, Silva and co-authors (Silva et al., 2015[39]) tested five newly synthesized thioxanthonic derivatives (TX1-5) in Caco-2 cells, observing that all examined compounds were able to promote both the induction and the activation of P-gp, although with a significantly more pronounced effect for TX5, with consequent lower cytotoxicity effects elicited by paraquat (Silva et al., 2015[39]). Our research group is committed to continue this line of research, aiming at finding P-gp inducers and/or activators at the intestine, with an adequate safety/efficacy relationship, using *ex vivo* and *in vivo* methodologies, in order to find enhancers of safety of xenobiotics. In this regard, TX5 showed to behave as a P-gp activator in the rat ileum (data submitted for publication). To accomplish our purpose, a first histological and toxicological evaluation of TX5 was performed, herein presented, testing two different doses administered to Wistar Han rats and selecting the less toxic dose to be used in the functional studies. 

## Materials and Methods

### Chemicals and drugs

TX5 hydrochloride was synthesized by the Organic and Pharmaceutical Chemistry Laboratory of the Faculty of Pharmacy of University of Porto, according to described procedures (Palmeira et al., 2012[28]). Briefly, a copper-catalyzed (CuI) cross-coupling between 1-chloro-4-propoxy-9*H*-thioxanthen-9-one and isobutylamine, in alkaline medium (K_2_CO_3_) and methanol, using a closed vessel and conventional heating (100 ^o^C), was performed. TX5 was characterized by spectroscopic methods and data was in accordance to described procedures (Palmeira et al., 2012[28]). The purity of the compound was determined by HPLC-DAD analysis, yielding a high degree of purity of at least 95 %. Bovine serum albumin (BSA), glycine, Tris hydrochloride, reduced glutathione (GSH), oxidized glutathione (GSSG), glutathione reductase (GR), 5,5′-dithiobis(2-nitrobenzoic acid) (DTNB), adenosine triphosphate (ATP), luciferase, D-luciferin, 2-vinylpyridine were all obtained from Sigma-Aldrich (Saint Louis, MI/USA). Folin-Ciocalteu reagent, copper (II) sulphate, perchloric acid (HClO_4_), ethylenediaminetetraacetic acid (EDTA), potassium bicarbonate (KHCO_3_), sodium hydroxide (NaOH), sodium carbonate, disodium phosphate, magnesium sulphate and potassium dihydrogen phosphate were purchased from Merck (Darmstadt, Germany). Potassium sodium tartrate was purchased from Fluka (Buchs SG, Switzerland). Sodium phosphate monobasic and reduced β-nicotinamide phosphate adenine dinucleotide (β-NADPH) were obtained from Panreac (Barcelona, Spain). Sodium chloride was purchased from VWR. Xylazine and ketamine were obtained from Novavet (Bragança, Portugal) and heparin from Braun (Germany). Isoflurane (Isoflo®) was purchased from Abbott (IL, USA). For histopathological analysis, commercial 4 % buffered formalin was acquired from Klinipath (Netherlands), while ethanol was purchased from Panreac AppliChem (Darmstadt, Germany), xylene from BDH - Prolabo, VWR International (Ireland), and paraffin from Merck (Germany). Chemicals for hematoxylin-eosin (H&E) and periodic acid-schiff (PAS)/Alcian Blue stains were purchased from Sigma-Aldrich (Saint Louis, MI/USA). All reagents used were of analytical grade or of the highest grade available.

### Animals

Male Wistar Han rats, weighing in average 240 ± 35 g, were born at the Institute of Biomedical Sciences Abel Salazar - University of Porto (ICBAS-UP) animal facilities. Animals were kept under light/dark cycles of 12/12 h, at 22 ± 2 °C room temperature and 50-60 % humidity and had access to water and pellet food *ad libitum*. Handling and care of animals were conducted according to the European Union guidelines for animal research (2010/63/EU) and the current Portuguese Law (Decreto-Lei no. 113/2013, de 7 de Agosto). Moreover, animal experiments were licensed by the Organism responsible for the welfare of animals (ORBEA, ICBAS-UP; protocol no. 250/2018). All procedures were carried out considering the compliance of 3 R's (replacement, reduction, refinement), providing appropriate animal care, minimizing their suffering and stress. 

### Experimental protocol

Before the experiment, animals were subjected to a short adaptation period to the metabolic cages, in order to reduce stress. Prior to TX5 or vehicle administration, animals were maintained in groups allowing social interactions. Two experiments were carried out, by using two distinct doses of TX5 hydrochloride (30 and 10 mg/kg, b.w.), administered by gavage. In the first experiment, a preliminary assessment of safety was performed by using four animals, two as controls and two treated with the highest dose of TX5 hydrochloride (30 mg/kg; TX5-30 group). In the second experiment, a lower dose of TX5 hydrochloride (10 mg/kg; TX5-10 group) was administered to perform the complete biochemical and histological evaluation, by using 14 animals (n = 7 controls; n = 7 TX5-treated). In both experiments, animals were fasted for 4 hours prior to gavage administration, while maintaining free access to water supplemented with 1 % sugar. Each animal was subjected to a brief inhalatory anesthesia with isoflurane to reduce the discomfort associated to the administration of the compound (or vehicle) by gavage. Immediately after the administration and for the next 24 hours, animals were individually housed in metabolic cages for whole urine collection. All animals were fasted for the last 12 hours before sacrifice, but water supplemented with 1 % sugar was given *ad libitum*. TX5 hydrochloride was prepared at the day of use in a concentration of 2 mg/mL in ultrapure water. A volume of 1 mL was administered *per* 200 g b.w. Control animals received ultrapure water using equivalent administration volumes of TX5-treated animals.

### Blood and urine collection and processing for hematological and biochemical analysis

Twenty-four hours after TX5/vehicle administration, rats were deeply anesthetized with a mixture of xylazine and ketamine [10 and 90 mg/kg b.w., intraperitoneally (i.p.), respectively]. Animals were placed in the *decubito supino* position and the abdomen was opened, exposing the caudal cava vein, from which blood was collected and placed into heparin-, citrate- and EDTA-containing tubes. The blood collected into heparinized and citrate-containing tubes was centrifuged (1670 x *g*, 15 min at room temperature) and the obtained plasma was stored at -80 °C for further biochemical and coagulation analysis, respectively. Whole blood samples (collected using EDTA as anticoagulant) were used for hematological evaluations, namely erythrocyte count, hemoglobin (Hb) concentration, hematocrit, hematological indexes - mean cell volume (MCV), mean cell hemoglobin (MCH), mean cell hemoglobin concentration (MCHC), red cell distribution width (RDW), platelet (PLT) count, plateletcrit (PCT), platelet distribution width (PDW), mean platelet volume (MPV), total and differential white blood cell (WBC) count, using an automated blood cell counter (Sysmex K1000, Hamburg, Germany). Blood smears were stained according to Wright (International Committee for Standardization in Haematology, 1984) and blood cell morphology was observed. For the coagulation study, the prothrombin time (PT) and the activated partial thromboplastin time (APTT) were evaluated by using the clotting assay (Coagulation analyzer Coatron M4, Teco Medical Instruments GmbH, Neufahrn, Germany; APTT-XL and TEClot PT-S, Teco Medical Instruments GmbH, Neufahrn, Germany).

The urine of each animal was collected 24 hours after TX5 or vehicle administration (metabolic cage) and centrifuged (3220 x *g*, 15 min at 4 °C). The resulting supernatants were separated and measured to assess the individual urinary flows (mL/day), and frozen at -80 °C for further analysis.

Plasma and urine samples were used to evaluate several clinical chemistry parameters on an AutoAnalyzer (PRESTIGE® 24i, PZ Cormay S.A.), using the respective kits and following the manufacturer's instructions. 

### Tissue collection and processing for histological and biochemical analysis

Immediately after exsanguination, the liver, kidneys, heart, spleen, lungs, intestine and brain were removed, washed in refrigerated saline solution (0.9 % NaCl), gently dried with filter paper and weighted to assess the relative organ weight (ROW, calculated as a percentage of the total body weight at the sacrifice day). Organs were then processed for histological and biochemical analysis.

### Histological analysis

After animal sacrifice, pieces of the heart, small intestine, liver, kidneys, lungs, spleen and brain were randomly selected and immediately fixed in commercial 4 % buffered formalin for histopathological evaluation. After a fixation period of 24 hours, tissues were dehydrated through a series of graded ethanol solutions (70.0 - 99.8 %), cleared in xylene and impregnated and embedded in paraffin. Each organ was sectioned (Microtome - Leica RM 2255, Germany) into thin sections (4 µm thick). In order to improve section adhesion, the sampled sections were mounted in silane-coated microscope slides [Nuova Aptaca, Canelli (AT), Italy]. The sections were then stained with H&E and PAS/Alcian Blue. For the histopathological analysis, the slides of all treatments were examined blinded and photographed under light microscope (BX50, Olympus, Japan) equipped with a digital camera (DP21, Olympus, Japan).

### Biochemical analysis

The remaining tissue samples were homogenized in ice-cold 25 mM phosphate-buffered solution [1:4 (m/v), Ultra-Turrax® Homogenizer], pH 7.4. The homogenates were kept on ice, then centrifuged at 3000 x *g* for 10 min, at 4 °C. An aliquot of the resulting supernatants was separated for total protein quantification, and stored at -20 °C. Another aliquot of the resulting supernatants was precipitated with an equal volume of 10 % HClO_4_ (5 % final acid concentration) and again centrifuged (16 000 x *g*, 10 min at 4 °C). Aliquots of the acidic supernatants were then separated and frozen at -80 °C for ATP quantification, and at -20 °C for total glutathione (tGSH) and oxidized glutathione (GSSG) quantification. 

#### Determination of tissue ATP levels

The ATP levels of tissue homogenates were determined by bioluminescence, which is based on the generation of light using the luciferin-luciferase system (Costa et al., 2007[7]; Rossato et al., 2014[32]). The method uses the enzyme luciferase that catalyzes the oxidation reaction of the luciferin reagent, consuming ATP. This reaction gives rise to light, being the amount of ATP present in the sample linearly proportional to the amount of light emitted. Briefly, 150 µL of the acidic supernatant referred in the section “Biochemical analysis” was neutralized with 150 µL of 0.76 M KHCO_3_ and centrifuged (16 000 x *g*, 1 min at 4 °C). Bioluminescence was immediately read in a microplate reader Biotech Synergy HT (Winooski, VT/USA) after the reaction of 100 μL of neutralized supernatant with an equal volume of luciferin/luciferase solution (prepared in luciferin/luciferase buffer: 50 mM glycine, 10 mM MgSO_4_, 1 mM Tris-HCl, 0.55 mM EDTA, 0.1 % BSA; pH 7.6). ATP standards were prepared in 5 % HClO_4 _and treated like the samples. ATP intracellular levels were expressed in nmol of ATP *per* mg of protein.

#### Determination of tissue total glutathione (tGSH) and oxidized glutathione (GSSG)

The tGSH and GSSG levels of tissue homogenates were determined by the DTNB-GSSG reductase recycling assay (Costa et al., 2007[7]; Teixeira-Gomes et al., 2016[45]). The method involves oxidation of GSH by the sulfhydryl reagent DTNB to form the yellow derivative 5'-thio-2-nitrobenzoic acid (TNB), measurable at 415 nm. This formation is proportional to the concentration of GSH in the sample. The GSSG is recycled to GSH by GR, in the presence of NADPH. For the quantification of GSSG, 2-vinylpyridine was used to block GSH. The levels of GSH were determined considering the following formula: GSH = tGSH - 2 × GSSG. Briefly, for tGSH quantification, 200 μL of the acidic supernatant referred in the section “Biochemical analysis” was neutralized with 200 μL of 0.76 M KHCO_3_ and centrifuged (16 000 x *g*, 2 min at 4 °C). For GSSG quantification, 10 μL of 2-vinylpyridine was added to 200 μL of acidic supernatant, and the samples were shaken during 1 hour on ice prior to the neutralization step with 0.76 M KHCO_3_. In 96-well plates, 100 μL of neutralized supernatant of sample, standard or blank were added in triplicate and mixed with 65 μL of fresh reagent solution containing 1.3 mM DTNB and 0.24 mM NADPH. Plates were incubated at 30 °C for 15 min in a plate reader (PowerWaveX; Bio-Tek Instruments, Winooski, VT/USA) prior to the addition of 40 μL GR solution (10 U/mL). The final product of this reaction is a colored substance, and its formation was monitored for 3 min, at 415 nm, and compared with a standard curve (Pontes et al., 2008[30]). GSH and GSSG standard solutions were prepared in 5 % HClO_4_ and treated like the samples. Levels of tGSH, GSSG and GSH were normalized to the protein content (results are presented in nmol tGSH *per* mg of protein or nmol GSSG *per* mg of protein or nmol GSH *per* mg of protein).

#### Determination of total protein tissue levels

Protein quantification was carried out according to the method previously described by Lowry et al. (1951[24]). An aliquot of the tissue homogenate supernatant referred in the section “Biochemical analysis” was used to assess the total tissue protein levels by spectrophotometry, using a microplate reader (wavelength = 750 nm). BSA standard curves were prepared in 0.5 M NaOH.

### Statistical analysis

Statistical analysis was performed with the GraphPad Prism software program version 6 (San Diego, CA/USA) and the results presented as means ± standard error of the mean (SEM). Outliers were identified using the ROUT test. The Shapiro-Wilk normality test was conducted before group comparison. Statistical comparison between control and TX5-exposed groups was estimated using the Unpaired Student *t*-test when the distribution was normal and the Mann-Whitney test when the distribution was not normal. A *p* value lower than 0.05 was considered to denote statistically significant differences.

## Results

### Organ weights were not changed by TX5

The individual weight of each organ (heart, liver, brain, kidneys, small intestine, spleen, lungs) was registered and the ratio organ weight/body weight (ROW) was calculated. Statistical analysis was only done for the TX5-10 group. No significant differences were observed between TX5-exposed and control animals for all collected organs (Table 1(Tab. 1)). Additionally, no differences were observed in the pattern of water intake and in the individual body weights between t = 0 h and t = 24 h (data not shown).

### TX5 administration induced extensive hepatic necrosis at 30 mg/kg, but this effect was not observed at 10 mg/kg 

**Figure 1:**
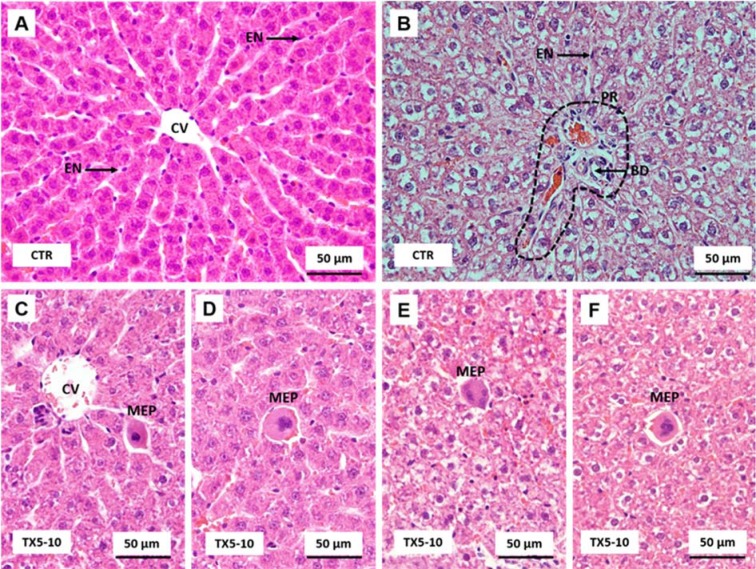
Light microscopy photomicrographs of H&E stained sections of different liver regions from control (A and B) and TX5-10-treated (C-F) rats. CV, central vein; EN, endothelial cell; BD, bile duct; PR, portal region (dashed line); MEP, megakaryocytic precursors. CTR - Control group; TX5-10 - thioxanthone 5, 10 mg/kg group

The morphology of the liver was very similar in animals from all groups. It was possible to observe the classic lobules defined as polygonal structures with several portal tracts at the periphery and a central vein in the center (Figure 1A(Fig. 1)). Hepatic cords or plates are composed of columns of hepatocytes extending from the portal region to the central vein. The spaces between the plates, seen as pale white regions, contain the liver sinusoids or “capillaries” of the liver. Due to the smaller size and decreased amount of connective tissue, portal regions may frequently be overlooked in histologic sections, being composed by four elements: arterioles, venules, bile ducts and lymphatics (Figure 1B(Fig. 1)). The main difference between the liver of control and TX5-10-exposed animals is the presence of a higher number of megakaryocytic cells in the animals exposed to TX5, denoting a reactive megakaryocytopoiesis (Figure 1C-F(Fig. 1)). 

**Figure 2:**
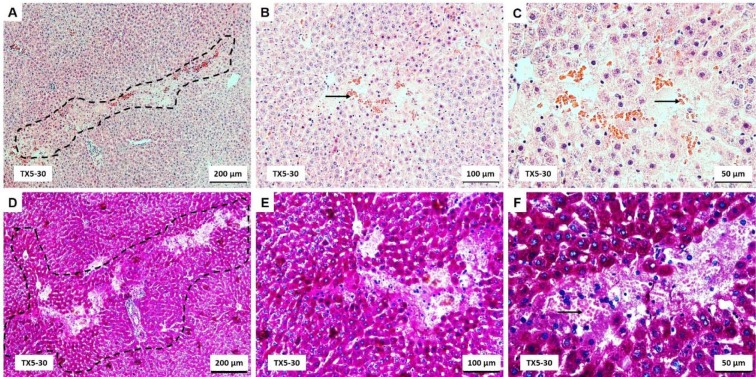
Light microscopy photomicrographs of H&E (A-C) and PAS/Alcian Blue (D-F) stained sections of the liver of TX5-30 group, showing confluent necrosis with central lobular hemorrhage (arrows) and an increase in hepatocytes glycoprotein content (dashed line). TX5-30 - thioxanthone 5, 30 mg/kg group

Regarding TX5-30 rats, extensive necrosis was observed (Figure 2(Fig. 2)). Intrahepatic glycogen and/or other glycoproteins were almost wholly concentrated in liver sections of TX5-exposed rats, mainly near the necrotic areas (Figure 2(Fig. 2)). These areas presented swelled cells, with clumping of nuclear chromatin and breaks in the plasma membrane. In small areas, the complete release of cellular components into the extracellular environment caused an inflammatory response. When both hepatocellular necrosis and destruction of the endothelial cells occurred, a central lobular hemorrhage into the zone of necrotic hepatocytes was observed.

### TX5 induced splenic parenchyma hyperemia at 30 mg/kg, but this effect was not observed at 10 mg/kg 

**Figure 3:**
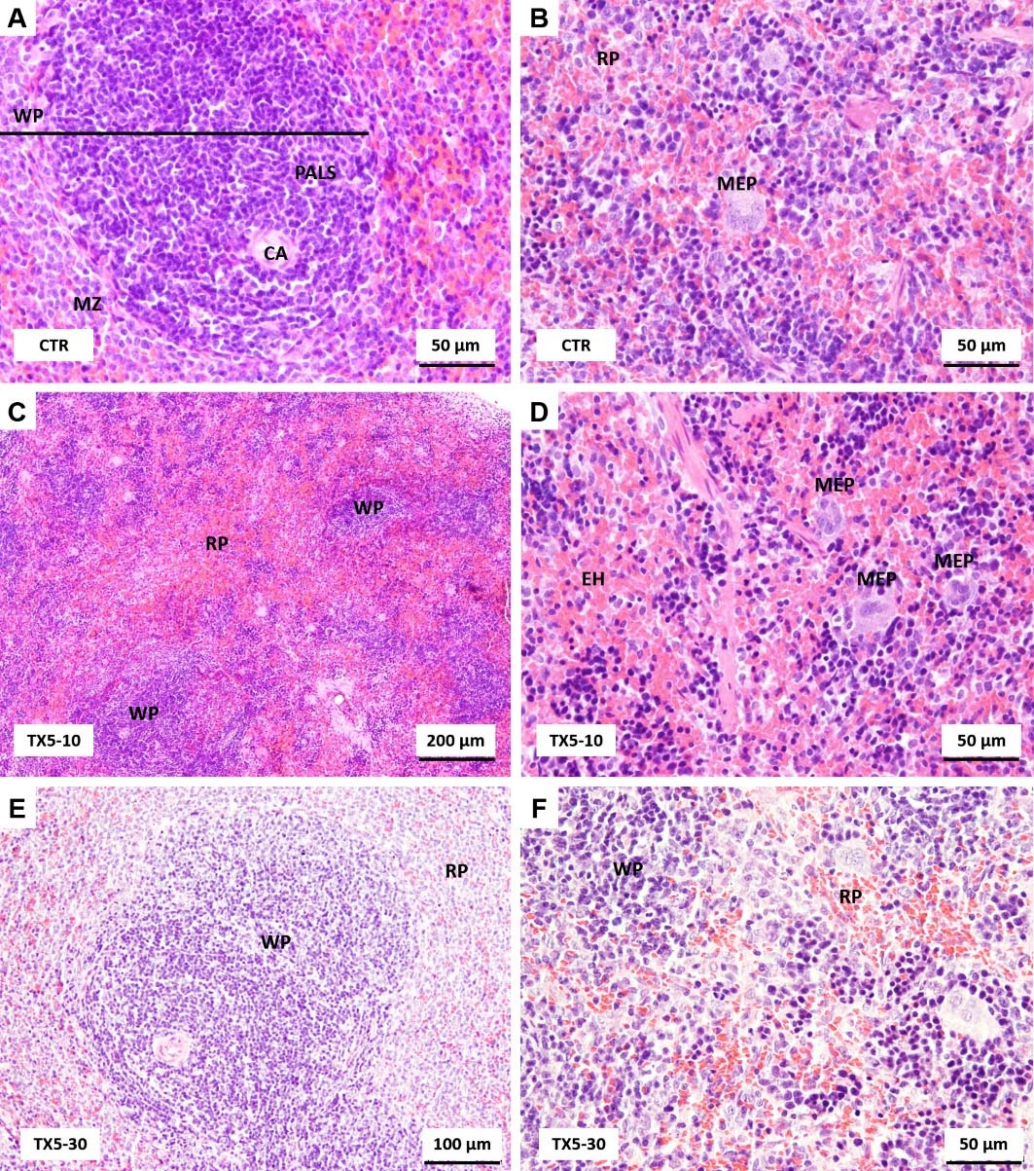
Light microscopy photomicrographs of H&E stained sections of the spleen of control (A and B), TX5-10-exposed (C and D) and TX5-30-exposed (E and F) rats. CA, central artery; EH, erythroid hyperplasia; MEP, megakaryocytic precursors; MZ, marginal zone; PALS, periarterial lymphatic sheath; RP, red pulp; WP, white pulp. CTR - Control group; TX5-10 - thioxanthone 5, 10 mg/kg group; TX5-30 - thioxanthone 5, 30 mg/kg group

The substance of the spleen is divided into white and red pulp. White pulp consists of a cylindrical mass of lymphocytes arranged around a central artery that constitutes the periarterial lymphatic sheath (PALS). Splenic nodules occur along the length of the PALS. When observed in cross section through part of the sheath that contains a nodule, the central artery appears eccentrically located with respect to the lymphatic mass. Red pulp consists of splenic sinuses surrounded by splenic cords (cords of Billroth). The splenic red pulp is a normal site of low levels of hematopoiesis and a major site of reactive hematopoiesis (*i.e.*, myeloid, erythroid and megakaryocytic hyperplasia). As in the liver, the main difference observed between control and TX5-10-exposed animals was a prominent megakaryocytic hyperplasia observed in the red pulp of the treated group (Figure 3C and D(Fig. 3)). 

The spleen tissue from TX5-30 group exhibited marked histological changes characterized by splenic parenchyma with hyperemia of red pulp (Figure 3E and F(Fig. 3)). Additionally, small hemorrhagic areas were observed in some sections. In these animals, the volume of both white and red pulp increased, being the increase in the splenic red pulp more pronounced than that of the white pulp. Additionally, it was observed a significant increase of the marginal zone of the follicles (Figure 3E and F(Fig. 3)).

### TX5 did not cause any change in the heart at both doses

**Figure 4:**
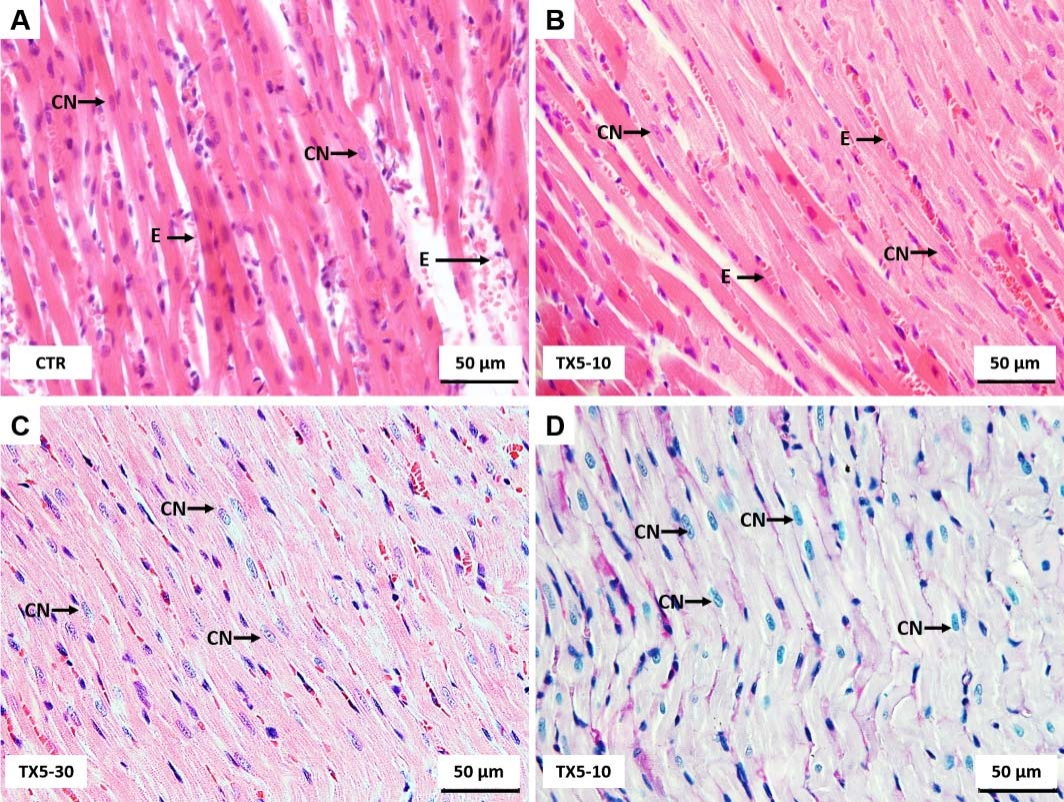
Light microscopy photomicrographs of H&E (A-C) and PAS/Alcian Blue (D) stained sections of myocardial tissue of control (A) and TX5-exposed (B-D) groups. CN, cardiomyocyte nuclei; E, erythrocytes. CTR - Control group; TX5-10 - thioxanthone 5, 10 mg/kg group; TX5-30 - thioxanthone 5, 30 mg/kg group

Photomicrographs of H&E stained sections of myocardial tissue revealed that myocardial cytoplasm and nuclei were clear and well distributed. In all groups, cardiomyocytes with eosinophilic cytoplasm occupy the largest myocardium. In Figure 4(Fig. 4), most muscular cells are oriented longitudinally, and the faint cross-striations are evident. Cardiomyocyte nuclei are large, with an ellipsoid shape, and contain granular chromatin with one or two nucleoli. Between cardiomyocytes it is observed loose connective tissue containing fibroblasts and capillaries lined by endothelial cells. Erythrocytes are present within capillaries. No visible changes in the total of glycoproteins were observed in the animals of both groups. There was a very slight, non-relevant, hyperemia in the myocardium of the TX5-30 animals (Figure 4C(Fig. 4)). No visible changes in the total of glycoproteins occurred in the animals of all groups (Figure 4D(Fig. 4) herein for TX5-10 group). 

### TX5 did not induce lung alterations

**Figure 5:**
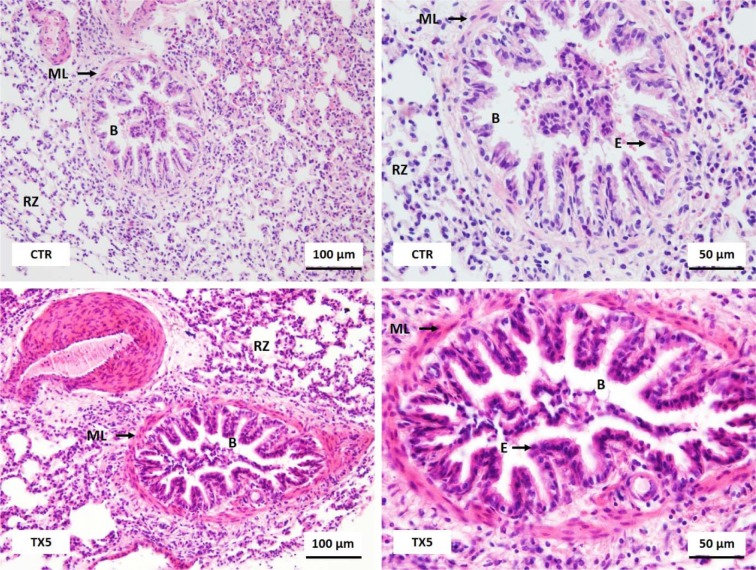
Light microscopy photomicrographs of H&E stained sections of the lungs of control (CTR) and TX5-exposed animals (10 mg/kg). B, bronchiole; RZ, respiratory zone; ML, muscular layer; E, epithelium

No morphological alterations were found in the lungs of control and TX5-exposed rats, at both doses. Figure 5(Fig. 5) is composed by photomicrographs of an intrapulmonary bronchiole, lined with columnar to cuboidal epithelium with some ciliated cells. The very thin *lamina propria *and submucosa separate epithelial cells from the muscular layer. The alveolar duct has a wall formed by alveoli and sparse bundles of smooth muscle. The alveolar sacs have a wall formed completely by alveolar septa. Alveoli emanating from neighboring alveolar ducts and respiratory sacs are connected through pores in the alveolar septa.

### TX5 caused minor morphological alterations in the small intestine at 30 mg/kg

**Figure 6:**
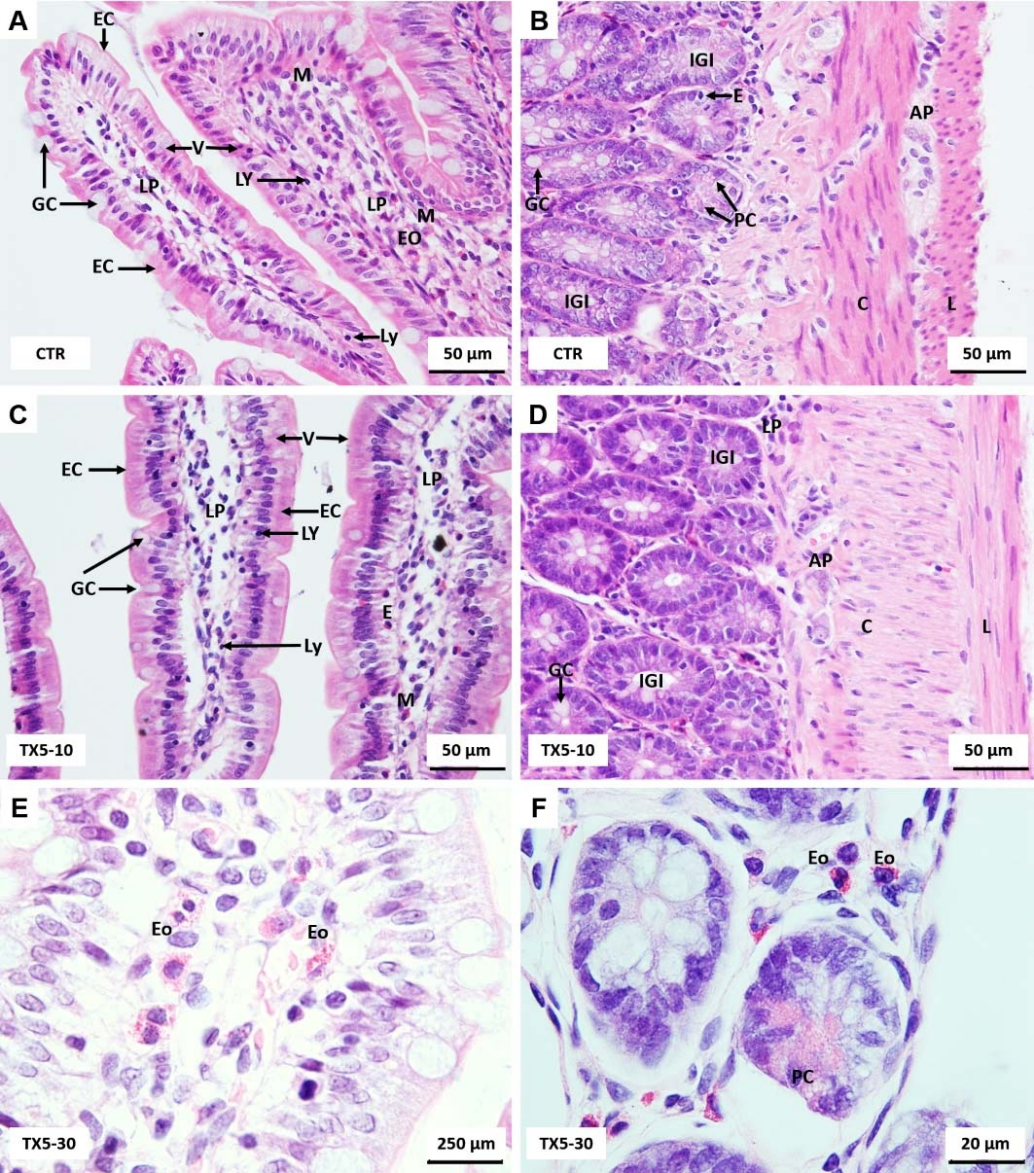
Light microscopy photomicrographs of H&E stained sections of the rat small intestine from control (A and B), TX5-10-treated (C and D) and TX5-30-treated (E and F) rats. AP, Auerbach's plexus; C, circular (inner) layer of *muscularis externa*; E, enteroendocrine; EC, enterocytes; Eo, eosinophils; GC, goblet cells; IGl, intestinal glands (crypts); L, longitudinal (outer) layer of *muscularis externa*; LP, *lamina propria*; LY, lymphocytes; M, mastocytes; PC, Paneth cells; V, villi. CTR - Control group; TX5-10 - thioxanthone 5, 10 mg/kg group; TX5-30 - thioxanthone 5, 30 mg/kg group

In the small intestine of all animals, the villous epithelium is contiguous with the crypt epithelium, which is composed of surface absorptive cells (enterocytes), goblet cells, stem cells, Paneth cells and enteroendocrine cells (Figure 6(Fig. 6)). Enterocytes are tall columnar cells with an apical (luminal) surface covered with closely packed microvilli (brush border). Goblet cells are responsible for producing mucin. Stem cells divide rapidly to replenish the epithelium. The exocrine serous Paneth cells have abundant brightly eosinophilic cytoplasmic granules within their cytoplasm. Enteroendocrine cells exist near the basement membrane. Intraepithelial lymphocytes are also common. Plasma cells, macrophages, eosinophils and mast cells are also present in the *lamina propria* of small intestine. The *muscularis externa* consists of two concentric and relatively thick layers of smooth muscle. The cells in the inner layer form a tight spiral, described as a circularly oriented layer; those in the outer layer form a loose spiral, described as a longitudinally oriented layer. Within the connective tissue located between the two-muscle layer lies the myenteric plexus (also called Auerbach's plexus), containing nerve cell bodies (ganglion cells), as well as blood vessels and lymphatic vessels (Figure 6(Fig. 6)). All these features are similarly observed in control and TX5-exposed animals. However, rats exposed to TX5, at both doses, apparently had an inflamed small intestine with the presence of a higher number of lymphocytes, eosinophils and mast cells, being in higher number in animals exposed to the higher dose (30 mg/kg) (Figure 6E and F(Fig. 6)).

**Figure 7:**
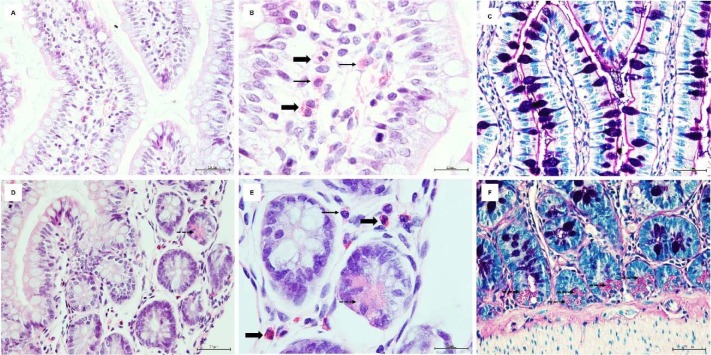
Photomicrographs of H&E (A, B, D, E) and PAS/Alcian Blue (C and F) stained sections of small intestine of TX5-30 group (thioxanthone 5, 30 mg/kg). Thin arrows - Mastocytes; Thick arrows - Eosinophils; Dashed arrow - Paneth cells

Rats exposed to TX5 apparently had an increase in Paneth cells number, located at the base of crypts of Lieberkühn. The Paneth cells of rats administered with TX5 at higher dose (30 mg/kg) also had more and bigger cytoplasmic granules that were PAS positive (Figure 7(Fig. 7)). Additional morphologic relevant alterations were not observed in the small intestine.

### TX5 caused a slight hyperemia and tubular edema in rat kidneys at 30 mg/kg

**Figure 8:**
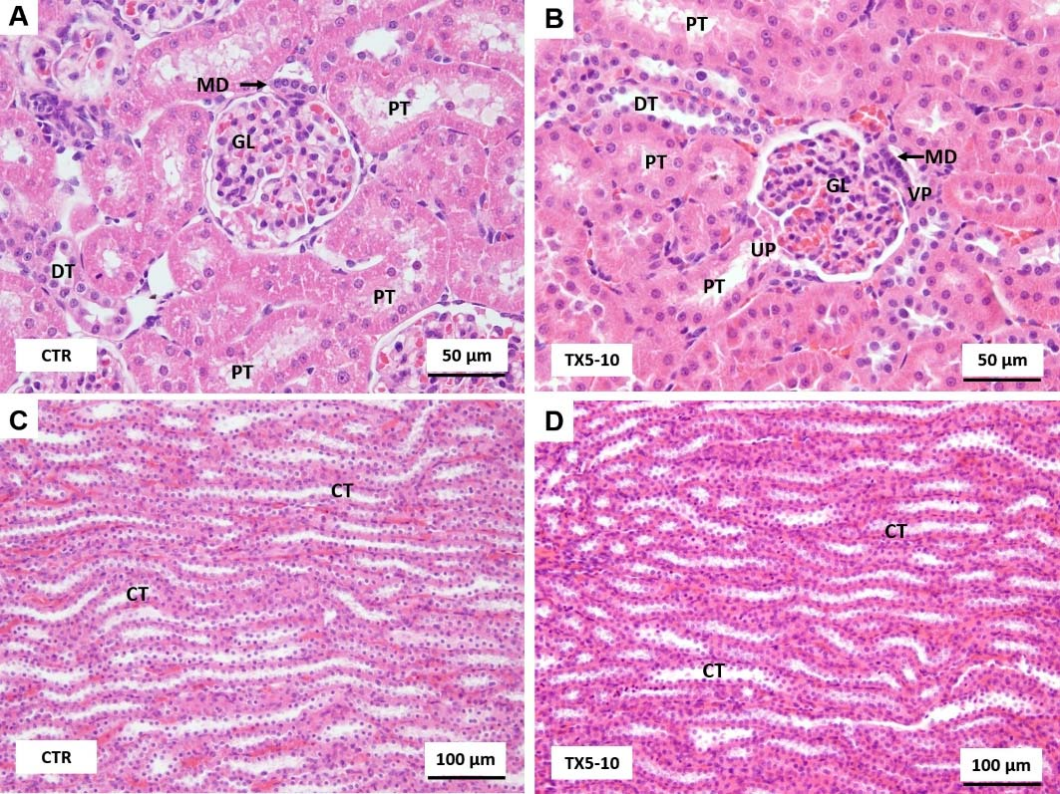
Light microscopy photomicrographs of H&E stained sections of the kidneys from control (A and C) and TX5-10-treated (B and D) rats. CT, collecting tubule; DT, distal tubule; GL, glomerulus; MD, macula densa; PT, proximal tubule; VP, vascular pole of renal corpuscle; UP, urinary pole of renal corpuscle. CTR - Control group; TX5-10 - thioxanthone 5, 10 mg/kg group

The kidney cortex consists of renal corpuscles along with the convoluted tubules and straight tubules of the nephron, the collecting tubules, collecting ducts, and an extensive vascular supply. The kidney medulla is characterized by straight tubules, collecting ducts and a special capillary network, the *vasa recta*. In the kidneys, normal glomerular and tubular histology was registered in animals of both control and TX5-10 group (Figure 8(Fig. 8)).

**Figure 9:**
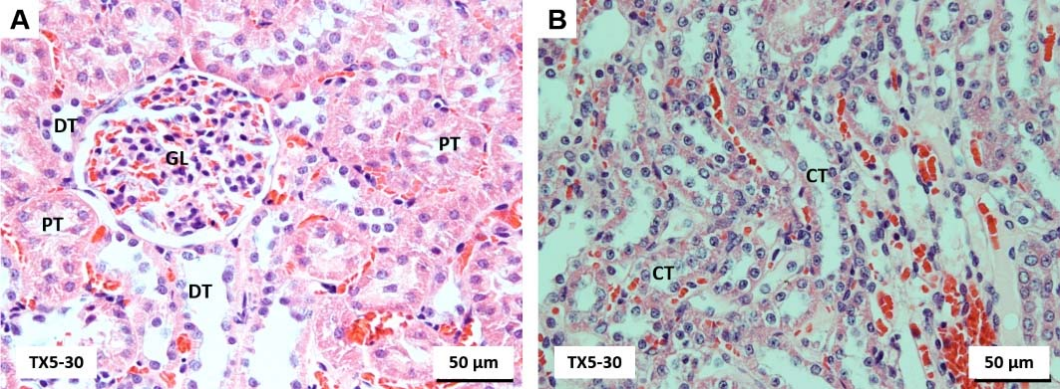
Light microscopy photomicrographs of H&E stained sections of the kidneys of TX5-30 animals group. It is possible to observe slight hyperemia (A, B) and tubular edema (B). CT, collecting tubule; DT, distal tubule; GL, glomerulus; PT, proximal tubule. TX5-30 - thioxanthone 5, 30 mg/kg group

On the contrary, the kidneys of TX5-30 rats presented a slight hyperemia (Figure 9A and B(Fig. 9)) and tubular edema (Figure 9B(Fig. 9)). 

### TX5 did not cause brain histopathological changes 

**Figure 10:**
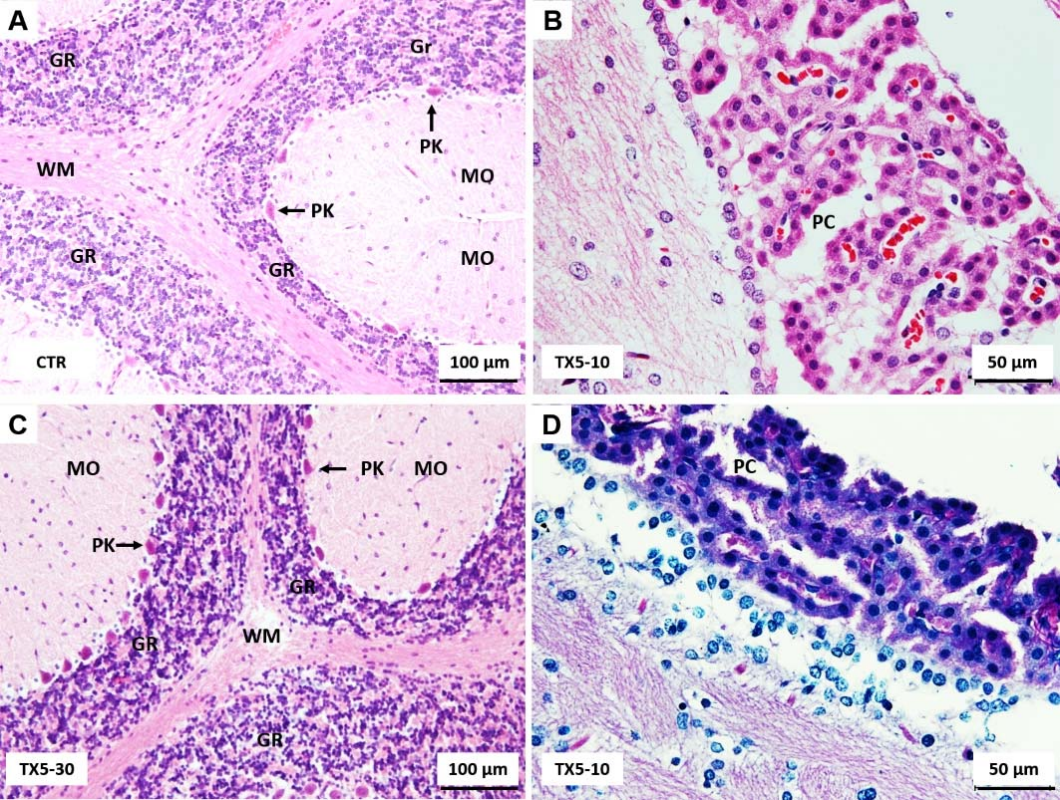
Light microscopy photomicrographs of H&E (A-C) and PAS/Alcian Blue (D) stained sections of the nervous tissue from control (A) and TX5-treated (B-D) rats. As an example, herein, the cerebellum (A and C) and the choroid plexus (B and D) are shown. No brain histopathological changes were observed among groups. GR, granular layer; MO, molecular layer; PK, Purkinje cell layer; WM, white matter; PC, Choroid plexus. CTR - Control group; TX5-10 - thioxanthone 5, 10 mg/kg group; TX5-30 - thioxanthone 5, 30 mg/kg group

Microscopic assessment is a well‐validated approach for evaluating nervous system structural abnormalities in rodents, providing an excellent correlation with gross pathology. In this study, the nervous system cytoarchitecture was normal in animals of all groups. All animal's hippocampus evidenced pyramidal neurons which were densely packed and of small size. Gray matter has cell bodies neurons associated with neuropil that is a meshwork of axonal, dendritic and glial processes (data not shown). No differences were observed in the cerebellum of control and TX5-exposed rats. The peripheral gray matter is uniformly organized in three layers, which are (from outside to inside) the molecular layer, Purkinje cell layer and the granular layer. The molecular layer is a broad expanse of densely packed neuronal processes with few neuronal bodies. The Purkinje cell layer is a monolayer of large, torpedo-shaped cells with prominent apical processes extending into the molecular layer. The granular layer is packed with small, dark, round granule cells and Golgi cells (Figure 10A and C(Fig. 10)). The choroid plexus, modified ependymal cells that cover the capillary loops and are responsible for the production of the cerebrospinal fluid (CSF), had normal appearance and contained the normal amount of glycoproteins, as revealed by PAS/Alcian Blue histochemistry (Figure 10D(Fig. 9)).

### No changes in both plasma and hematological parameters, but a higher tendency to blood coagulation after TX5 administration

Given the evident histological alterations observed in the rats administered with TX5 30 mg/kg in the pilot assay, we continued this study by electing the lower dose tested (10 mg/kg) for which, as mentioned before, no important histopathological changes were observed in any evaluated organs, with the exception of a moderate megacaryocytic hyperplasia in both the liver and the spleen. TX5 (10 mg/kg) did not induce any alteration in either the plasma biochemical or the hematological parameters evaluated (Table 2(Tab. 2)). Several parameters were measured as biomarkers of toxic effect. Particularly, the plasma levels of AST, ALT, CK, CK-MB, creatinine and urea were measured 24 hours after exposure to TX5 (10 mg/kg) as biomarkers of liver, heart, muscle or kidneys integrity. There were no significant differences in any of these parameters between control and TX5-exposed rats. Moreover, the AST/ALT ratio was also calculated and no differences were found (data not shown). Very importantly, TX5 was able to cause a significant decrease in APTT when compared to control animals (14.4 ± 0.4 seconds *versus* 18.3 ± 0.5 seconds, respectively), without alterations in PT, as shown in Table 2(Tab. 2).

Additionally, in the urine, as can be observed in Table 3(Tab. 3), no differences were observed in the biochemical parameters evaluated between control and TX5-exposed rats, with the exception of creatinine and sodium, both of which suffered a TX5-induced increase relatively to control animals (35.96 ± 3.93 *versus* 21.97 ± 3.43 mg/kg/day and 1.95 ± 0.31 *versus* 1.13 ± 0.19 nmol/kg/day, respectively). 

### TX5 increased ATP content in the small intestine

**Figure 11:**
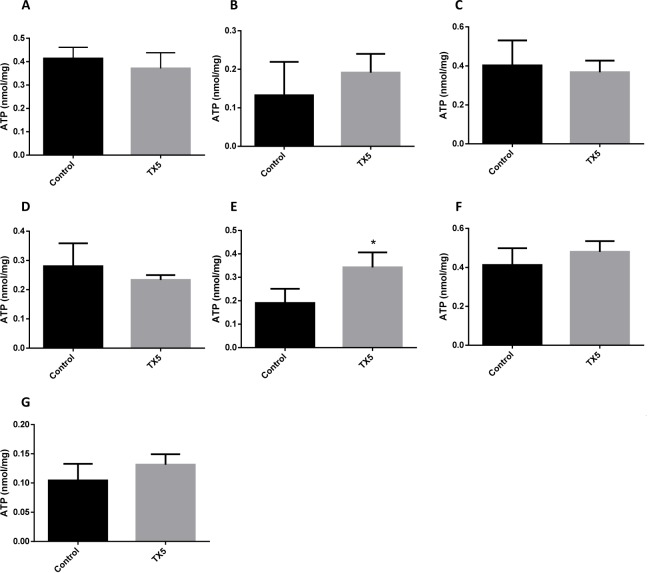
ATP levels in the heart (A), liver (B), brain (C), kidneys (D), intestine (E), spleen (F) and lungs (G) after TX5 (10 mg/kg) administration. Results, in nmol *per* mg of protein (nmol/mg), are presented as means ± standard error of the mean (SEM) and were obtained from six to seven animals in each group. Statistical comparisons were made using the Unpaired Student* t-*test (*p <0.05 TX5 *vs.* control).

As can be observed in Figure 11(Fig. 11) and Table 4(Tab. 4), TX5 significantly increased the ATP levels in the small intestine (0.34 ± 0.06 nmol/mg of protein) relatively to the control animals (0.19 ± 0.02 nmol/mg of protein), 24 hours after administration of the compound. No other changes in ATP content were evoked by TX5 in any of the other organs evaluated.

## Discussion

The history related to TXs is already long. The last twenty years were rich in disclosing new and challenging therapeutic applications for these compounds (Paiva et al., 2013[27]). Among them, TX5, a novel aminated thioxanthonic compound, has being deserved our attention in recent years, since docking studies uncovered its potential as a P-gp modulating agent (Palmeira et al., 2012[28]). Moreover, according to our previous data, TX5 showed to be a P-gp inducer and activator *in vitro,* in Caco-2 cells (Silva et al., 2015[39][41]), as well as *ex vivo*, using the rat ileum as a model (data submitted for publication). Based on these findings, the present study aimed at performing a preliminary *in vivo* safety study. It consisted in a first experiment in which a few number of Wistar Han rats (n = 2 controls and n = 2 TX5-treated) were administered, by gavage, with TX5 30 mg/kg b.w. This dose was selected according to a previous *in vivo* pharmacokinetic study (submitted elsewhere). During the 24 hours of the protocol, both putative behavior changes and the pattern of water and food ingestion were monitored and no alterations were noted. No differences were registered in the ROW, but it is not feasible to take strong conclusions given the low number of animals used in this first approach. In order to evaluate the degree of tissue damage due to TX5 treatment, we monitored tissue sections using microscopy. The histopathological analysis performed in the several organs (heart, liver, spleen, kidneys, small intestine, lungs and brain) revealed toxicity, particularly evident in the liver and spleen. We observed extensive hepatic necrosis (Figure 2(Fig. 2)), as well as hyperemia of the splenic parenchyma (Figure 3E and F(Fig. 3)). Moreover, signs of red pulp hemorrhage were observed in some sections after TX5 administration. Although with a mild degree of expression, it was observed a slight hyperemia in the myocardium of the animals exposed to TX5 (30 mg/kg), as well as an inflamed small intestine revealed by the presence of a higher number of lymphocytes, eosinophils and mast cells. Besides, the kidneys of the animals exposed to this dose of TX5 presented tubular edema and a slight hyperemia. On the contrary, there were no deleterious histological effects in the brain and the lungs. Given the impact of the histopathological analysis in the safety assessment of compounds, we considered these effects to be relevant. Consequently, the study was proceeded by testing a lower dose of TX5. 

Thus, in the second experiment, seven animals were administered with 10 mg/kg dose under the same protocol described for the highest dose tested. No differences were registered neither in the ROW nor in the water ingestion pattern. In histopathological terms, no relevant alterations were observed in any of the organs analyzed. Only a minor inflammation in the small intestine seemed to be present given the small number of inflammatory cells observed. Noteworthy, the necrotic effect in the liver observed with TX5 30 mg/kg was no longer observed with TX5 10 mg/kg. Additionally, at the lowest dose tested, the splenic toxic effects were not observed. 

The bone marrow is the major hematopoietic organ and a primary lymphoid organ responsible for the production of red cells, leukocytes and platelets, in response to systemic needs (Travlos, 2006[46]). However, it is known that extramedullary hematopoiesis occurs physiologically in the juvenile rat, presenting a tendency to decrease gradually with age and increasing in response to environmental stimuli (Figueiredo et al., 2016[13]; Parker and Papenfuss, 2016[29]). In the rat and the mice, extramedullary hematopoiesis has been recorded in the spleen, liver, lymphnode, kidney and adrenals (Raval et al., 2014[31]). Hematopoiesis in juvenile rats occurs primarily in liver and spleen. Particularly, it is common in rodent red pulp, especially in fetal and neonatal animals. Any combination of erythroid, myeloid and megakaryocytic cells may be evident (Cesta, 2006[5]; Parker and Papenfuss, 2016[29]). The histopathological analysis revealed the presence of megakaryocytopoiesis in the liver and in the spleen red pulp of both control and TX5-treated rats. Nonetheless and very interestingly, our qualitative analysis showed an apparent more prominent megakaryocytopoiesis in the liver and spleen of the animals exposed to TX5 (10 mg/kg) when compared to control rats. As stated before, extramedullary hematopoiesis can be increased as a consequence of xenobiotic's treatment (Domingues et al., 2011[11]; Mahiout et al., 2017[25]), an aspect that is in accordance with our findings. No signs of necrosis, lymphocytic infiltrate or hemorrhage were found in TX5-administered animals (10 mg/kg), in any of the other organs analyzed.

By the exposed, comparing the two doses tested, the histopathological findings showed that the lowest dose was apparently safe. As such, hematological and biochemical analysis were then performed. Indeed, the qualitative histological analysis only gives an indication of the degree of toxicity and should be combined with biochemical data to ascertain the overall toxicity induced by the compound. Alternatively, whenever changes are slight, quantification should be carried out so that they can be used, by themselves, in the conclusion of the observed results. 

The investigation of clinical pathology parameters (hematology, clinical chemistry and coagulation) is an important part of the preclinical evaluation of drug safety (Seibel et al., 2010[34]). In the present study, none of the hematological and biochemical parameters determined in the plasma of the animals showed any change due to exposure to TX5 (10 mg/kg). 

We have also evaluated the possible interference of TX5 on blood coagulation. Hemostasis is a cell-based process that is regulated in a tissue-specific manner by the differential expression of pro-coagulant and anticoagulant factors on endothelial cells from different sites throughout the vasculature (Fager and Hoffman, 2018[12]). Regarding coagulation, PT, APTT and thrombin time (TT) are the most commonly used clotting time assays in mammals. In fact, APTT and PT are the recommended laboratory tests of hemostasis for non-clinical toxicity and safety studies (Weingand et al., 1992[48]). PT, APTT and TT assess the function of the extrinsic, the intrinsic and the common pathways (Takahashi et al., 2011[44]). APTT can be used to evaluate the intrinsic and common pathways of coagulation and may be prolonged due to anticoagulant therapy, clotting factor deficiencies, lupus anticoagulant or acquired inhibitors of specific clotting factors (Loizou et al., 2018[23]; Winter et al., 2017[50]). Furthermore, it is known that there are differences in the time required for coagulation between species (Tabata et al., 1995[43]). Moreover, there are gender intra-species differences in the hemostatic parameters PT, APTT, TT and fibrinogen (Lemini et al., 2007[19]). In the rat, blood coagulation is faster than that in humans. On the other hand, according to Seibel and colleagues (2010[34]), the coagulation parameters might be susceptible to the sampling method used. Specifically, the authors compared PT and APTT from Sprague-Dawley blood samples collected terminally from the vena cava immediately before necropsy with samples taken from the sublingual vein also prior to necropsy. The results showed that APTT was significantly decreased in blood taken from the vena cava than that taken from the sublingual vein (Seibel et al., 2010[34]). In the present study, the values obtained for APTT in control rats were in accordance to those reported by Seibel et al. for the same vascular territory (Seibel et al., 2010[34]). Additionally, the APTT and PT values in the present study are in line with those presented by Lemini and colleagues, who studied the influence of species and gender in coagulation tests (Lemini et al., 2007[19]). Interestingly, according to our results, TX5 seems to accelerate blood coagulation. This pro-coagulant effect is revealed by a significantly reduction in the APTT of the rats exposed to TX5, when compared to the controls. Since no alterations were observed in PT, we hypothesize that the hemostatic effect is due to a change in the coagulation factors belonging to the intrinsic pathway, namely in factors VIII, IX, XI and/or XII. This TX5-mediated decrease of APTT may have implications in hemostasis management. Particularly, TX5 could confer protection against bleeding, at the potential expense of increased thrombotic risk. 

The kidney normally functions to maintain hemodynamic homeostasis and is a major site of damage caused by xenobiotic toxicity (Fisch et al., 2016[14]). Acute kidney injury is a heterogeneous group of conditions characterized by a sudden decrease in glomerular filtration rate (GFR), manifested by an increase in serum creatinine concentration or oliguria, and can be a consequence of drug toxicity (Levey and James, 2017[20]). Several urine biochemical parameters were measured to assess whether TX5 was able to induce renal toxicity, under the experimental conditions. Differently to the plasma biochemical results obtained, TX5 was able to significantly increase urinary creatinine and sodium. Creatinine is the final product of creatine phosphate catabolism. It is excreted in the kidneys via glomerular filtration, being a measure of GFR and a biomarker of renal injury (Wyss and Kaddurah-Daouk, 2000[51]). The increase in serum and urine creatinine was considered to represent an index of cisplatin-induced nephrotoxicity in Wistar rats, in such a way that the reversion of the toxic effect occurs through the normalization of these values (Khairnar et al., 2019[18]). Therefore, the urinary creatinine result may indicate some change at the renal level without a concomitant increase in plasma creatinine. In our study, plasma and urine urea levels were not altered by TX5. Similarly to our results, Lima and co-authors have shown that treatment with TXA1 hydrochloride (50 mg/kg, by subcutaneous injection 3 times per week), a TX5-related thioxanthonic compound, did not induce any change in both serum creatinine and urea of nude xenografted mice, 22 days after first treatment (Lima et al., 2018[21]). However, TXA1 hydrochloride was able to cause an alteration in steroid biosynthesis and an abnormal cellular cholesterol localization, both *in vitro* and *in vivo* (Lima et al., 2018[21]). The elevation in urinary creatinine observed in the present study would suggest the presence of histological alterations in the kidneys, which, however, were not observed. This is a very important conclusion in terms of a preliminary toxicity evaluation. In fact, a normal glomerular and tubular histology were seen in animals of both control and TX5-10 groups. Renal histopathological abnormalities were observed only at the highest dose (30 mg/kg) and were manifested by mild hyperemia and tubular edema. Additionally, TX5-10 rats presented a higher amount of sodium in urine when comparing to control animals. However, this effect was not accompanied by a concomitant increase in urinary volumes due to the osmotic effect of the electrolyte. We hypothesize that these results may indicate the ability of TX5 to alter sodium homeostasis, particularly by interfering with sodium reabsorption at tubular level. Nonetheless, the increase in sodium excretion might be a beneficial effect of the compound for certain pathological conditions. In fact, a similar effect was reported by other authors while studying antidiabetic agents and their impact in cardiovascular outcomes. In this regard, Lin and colleagues (2014[22]) reported that empagliflozin (*per os*, with standard diet containing empagliflozin 0.03 %), an inhibitor of the renal sodium/glucose co-transporter 2 (SGLT2) located at the apical membrane of the renal tubular epithelial cells, was able to significantly increase renal sodium excretion in the db/db mice diabetic model, on day 1 of treatment (Lin et al., 2014[22]). Although our results require further investigation, the possibility of TX5 being able to positively interfere with sodium homeostasis appears to be of great importance in the cardiovascular context.

We also tried to understand if TX5 could possibly cause toxicity by interfering with energetic metabolism and/or oxidative stress. Our study shows that TX5 did not cause significant alterations in the glutathione levels (tGSH, GSSG, GSH) of the several organs analyzed. Only an increase in the GSH/GSSG ratio was observed in the lungs of the rats exposed to TX5. This can be an indication of an interference of TX5 in the antioxidant defenses in this organ. The energetic metabolism was altered by TX5, with a significant increase in ATP levels, an effect observed exclusively in the small intestine of TX5-exposed animals (10 mg/kg). This effect might be related to an impairment in the bioenergetics in this organ induced by TX5. The lack of *in vitro* and *in vivo* data in the literature related to the interference of thioxanthonic compounds in bioenergetics and oxidative stress processes turns the discussion of our results more difficult. It is known that ATP bioavailability is related to mitochondrial function (Burke, 2017[4]). For example, one important mechanism of cytotoxicity relies on the induction of the mitochondrial permeability transition (MPT), which causes mitochondrial failure, leading to necrosis from ATP depletion or caspase-dependent apoptosis if ATP depletion does not occur fully (Jaeschke et al., 2002[17]). In this regard, several studies report ATP depletion as a consequence of xenobiotic-induced mitochondrial disruption in distinct tissues, such as the liver, heart and brain (Barbosa et al., 2015[2]; Rossato et al., 2014[32][33]; Shi et al., 2018[35]; Shiba et al., 2011[36]). In contrast, we found an increase in the intracellular concentration of ATP in the intestine of TX5-treated animals. Although, in the present study, mitochondrial morphologic changes were not observed in the intestine, we cannot exclude the existence of functional mitochondrial disturbances that could putatively explain the changes in ATP intestinal content found in TX5-exposed animals. This finding requires further research. We hypothesize that the increase in intestinal ATP might be related with the mechanism of action of TX5, *i.e.*, the TX5-mediated P-gp activator effect observed in the rat intestinal mucosa *ex vivo* (results submitted elsewhere). In fact, P-gp is an energy-dependent efflux protein, so its exacerbated activity induced by TX5 requires a greater supply of ATP and, consequently, the increase in ATP content observed in this study. Following this logic, there are studies reporting a decrease in tissue ATP levels associated with a reduction in the activity of ATP-dependent efflux carrier proteins in rats (Aleo et al., 2014[1]; Slater and Delaney, 1970[42]) and humans (Chijiiwa et al., 2002[6]). These effects, in turn, might be related to alterations in enzymatic activity, such as ATP synthase or ATPase. Indeed, ATP synthase is a central enzyme of the cellular energy metabolism and could constitute a target in reprogramming energetic metabolism (Bianchi et al., 2018[3]). Alternatively, the increase in ATP content observed in the intestine of TX5-treated rats could be due to an involvement in the inflammatory process observed, revealed by the presence of a higher number of lymphocytes, eosinophils and mast cells (Figure 6(Fig. 6)). Indeed, other authors have demonstrated that ATP influences the response of intestinal epithelial cells to TLR ligands and favors the maturation of dendritic cells to become inflammatory (Yao et al., 2012[52]). Another explanation might be the ability of TX5 to induce transient changes in ATP intestine levels, with an increase after a short period of exposure, followed by a return to normal levels after a longer exposure period. Accordingly, transient changes in ATP brain levels following amphetamines exposure, namely MDMA, were reported, although, in these cases, with a decrease after a short contact and a long-term returning to control values (Barbosa et al., 2015[2]; Teixeira-Gomes et al., 2016[45]). As previously stated, the TX5-induced increase in intestinal ATP content found in the present study deserves further investigation in order to unveil the possible mechanisms underlying this finding.

In summary, this study highlights the: 1) presence of relevant histological hepatic and splenic toxicity induced by TX5 (30 mg/kg); 2) absence of histological damage with TX5 (10 mg/kg); 3) APTT decrease in TX5 (10 mg/kg) animals; 4) creatinine and sodium increase in the urine of TX5 (10 mg/kg) rats; 5) increased ATP levels in the small intestine of TX5 (10 mg/kg) group; 6) absence of relevant oxidative stress deregulation in all studied organs.

Taken together, our results show that TX5, at a 10 mg/kg dose, does not induce significant toxicity in the several biological matrices studied but may possibly have a pro-coagulant effect. The renal and intestinal effects observed require more research, although do not constitute a source of relevant toxicity. 

This study encourages further investigation of this molecule considering its activator effect, already demonstrated by us, of the important P-gp efflux transporter at the rat intestinal mucosa, *ex vivo*. This finding brings us closer to the use of this compound, with an adequate safety/efficacy relationship, as an antidote or therapeutic adjuvant in the pharmacological approach to intoxications caused by P-gp substrates.

## Notes

Carolina Rocha-Pereira and Fernando Remião (UCIBIO/REQUIMTE, Laboratório de Toxicologia, Departamento de Ciências Biológicas, Faculdade de Farmácia, Universidade do Porto, Rua Jorge Viterbo Ferreira, 228, 4050-313 Porto, Portugal; eMail: remiao@ff.up.pt) contributed equally as corresponding authors.

## Funding

This research was developed under the Project NORTE-01-0145-FEDER-000024, supported by Norte Portugal Regional Operational Programme (NORTE2020), under the PORTUGAL 2020 Partnership Agreement (DESignBIOtecHealth-New Technologies for three Health Challenges of Modern Societies: Diabetes, Drug Abuse and Kidney Diseases) and Project No. POCI-01-0145-FEDER-028736, co-financed by COMPETE 2020, Portugal 2020 and the European Union through the ERDF, and by FCT through national funds. This work was also supported by UID/MULTI/04378/2019 with funding from FCT/MCTES, through national funds.

## Figures and Tables

**Table 1:**
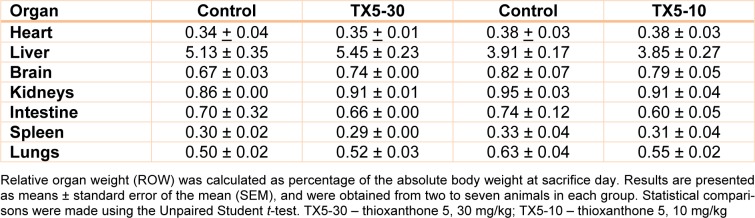
Relative organ weight (ROW) of control and TX5-exposed animals

**Table 2:**
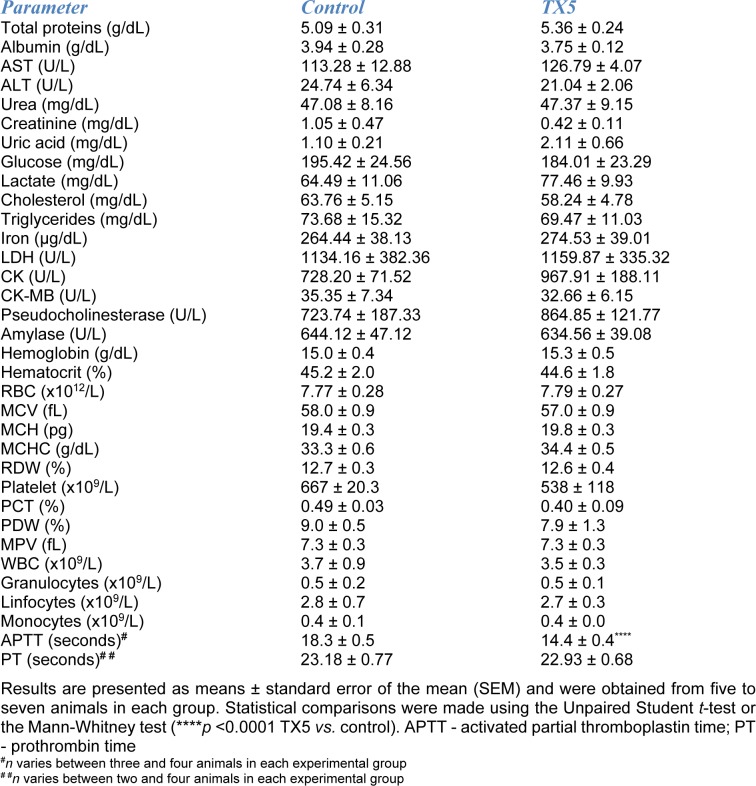
Plasma and hematological parameters of control and TX5-exposed (10 mg/kg) animals

**Table 3:**
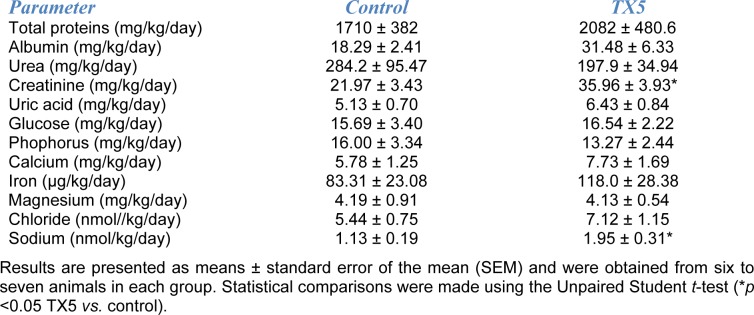
Urine biochemical parameters of control and TX5-exposed (10 mg/kg) animals

**Table 4:**
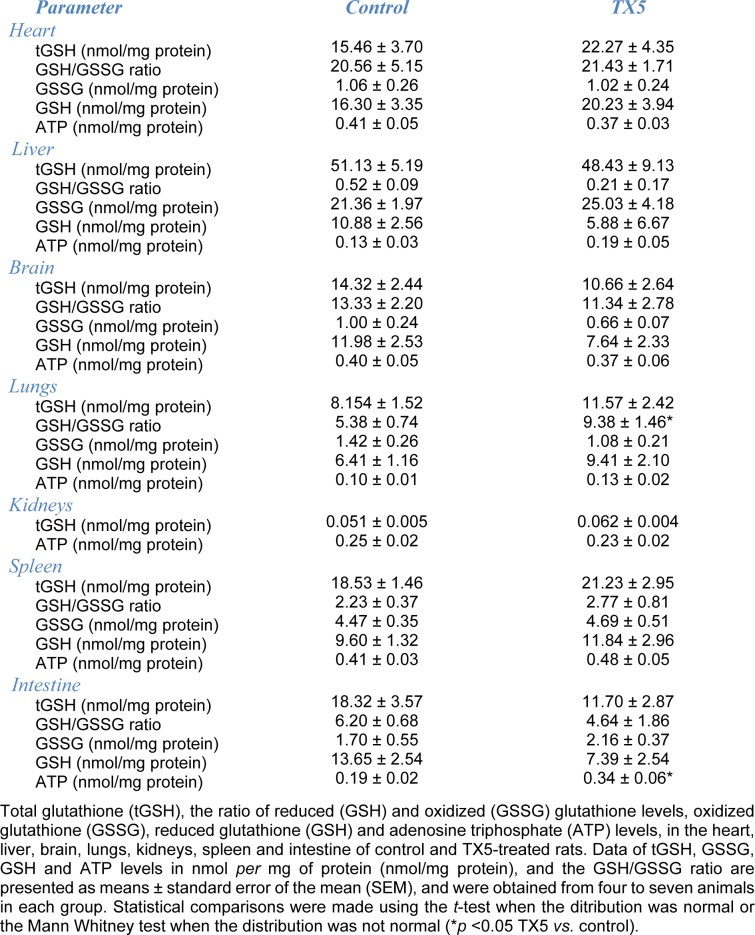
Effect of TX5 (10 mg/kg) administration in oxidative stress-related parameters and ATP levels in the seven evaluated organs
